# Why the simplest explanation isn’t always the best

**DOI:** 10.1073/pnas.2319169120

**Published:** 2023-12-20

**Authors:** Eva L. Dyer, Konrad Kording

**Affiliations:** ^a^Department of Biomedical Engineering, Georgia Institute of Technology, Atlanta, GA 30332; ^b^Department of Bioengineering, University of Pennsylvania, Philadelphia, PA 19104

As datasets in neuroscience increase in size and complexity, interpreting these high-dimensional data is becoming more critical. However, developing an intuition for patterns or structures in such datasets is hard. Dimensionality reduction methods aim to find patterns in these high-dimensional datasets, sometimes transforming them into simpler and more “interpretable” descriptions of the data (1). However, as Shinn in PNAS (2) underscores, what feels intuitive and simple can often mislead: Dimensionality reduction optimizes for specific statistical features of the data and doesn’t always agree with the most intuitive explanation.

Principal components analysis (PCA) is one of the most widely used dimensionality reduction techniques due to its conceptual simplicity and utility in data interpretation. The first principal component is defined as the direction of unit length that captures the maximum variance in the data, and the second principal component is the direction, orthogonal to the first, that captures the maximum remaining variance. This process continues for additional components, each capturing the maximum variance under the constraint of orthogonality to the previous components. When finished, this approach builds an orthogonal basis which constrains the collection of generator elements to have a particular geometry (Fig. 1). The principal components describe the data, but depending on the structure of the data, they may not align with the generating factors in data or, alternatively, produce hallucinated structure.

**Fig. 1.**
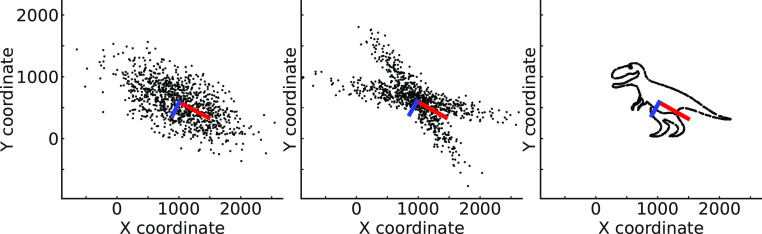
PCA can see structure that does not exist and miss structure that exists. All these datasets have the same principal components. (*Left*) if the data are Gaussian, then PCA is the ideal technique, extracting all the structure that is there. (*Middle*) when data are not Gaussian, PCA may “see” dimensions that do not exist, in this case stemming from there being multiple Gaussians. In such a case, relaxing the assumption of orthogonality could allow a model to extract the relevant aspects. (*Right*) when data are highly structured but not simple (also see ref. 3), PCA will not discover the relevant structure, but will see structure that is in a way not even there. Indeed, in this case of a single line graph another technique, such as isomap, would discover that the whole dinosaur is just a single line, or a 1D-manifold embedded in 2D.

As a compelling example of such “hallucinations,” Shinn (2) shows what happens when applying PCA to temporally or spatially smooth and shifted time-limited signals that are often encountered in neuroscience and behavioral analysis. In such cases, oscillatory PCs arise, due to smoothness either in time or space. Indeed, some of the oldest normative insights into neural coding came from the realization that PCA run on spatial low-pass signals (images) produces Fourier components (4). Just as a low-pass filter in Fourier analysis emphasizes the smooth, low-frequency components and simplifies the signal by excluding higher frequencies, PCA similarly discovers an oscillatory basis set when looking for an efficient basis for smooth signals. However, this similarity also highlights a critical interpretive hazard: just as many different signals can share similar low-frequency profiles, diverse datasets can yield similar principal components, complicating the attribution of specific meanings or origins to these components.

## Relaxing the Assumptions of PCA and Building More “Expressive” Models

While perhaps counterintuitive, more complex or expressive models that better align with the data's true generative structures offer a pathway to greater interpretability. For instance, using convolutional operations introduces shift invariance, enabling the model to recognize patterns irrespective of their position in the input space. This in turn can lead to more meaningful feature extraction and robust prediction. Moreover, relaxing constraints such as linearity and orthogonality can allow for more natural data representations. For example, independent component analysis (5) introduces the concept that natural data can often be represented as a combination of elements from an overcomplete dictionary, leading to representations that are both efficient and interpretable due to their parsimony (Fig. 1, *Middle*). Similarly in image processing, nonnegative matrix factorization (6) avoids the cancelation of positive and negative values, producing parts-based representations that can be more readily understood and interpreted by humans.

As Shinn in PNAS underscores, what feels intuitive and simple can often mislead: dimensionality reduction optimizes for specific statistical features of the data, and doesn’t always agree with the most intuitive explanation.

In neuroscience, there has been a push to build more expressive models of neural dynamics that can better capture the nonlinear structures present in large recordings (7). For instance, models like gaussian process factor analysis can be used to impose smooth structure on the generating factors (8), and jPCA can be used to encourage a set of dynamics that use oscillatory factors (9). Latent factor analysis through dynamics assumes a simple dynamical system model on a learned latent space (10). Contrastive learning methods take a different tact: Rather than trying to learn factors that can generate data, they instead learn embeddings of the data that are invariant to different transformations of the input (11–13). By defining positive and negative examples, these methods allow us to guide the model to focus on specific features in the data. All these methods allow us to detect highly nontrivial structure in datasets that may be missed using simpler linear models like PCA.

All dimensionality reduction techniques look for projections of data that are special in a way, just like PCA is looking for data that best explains the variance in the training data’s distribution. However, as generative models become more expressive and overparameterized, they can also become less interpretable and can overfit specific nuances in the data. For example, jPCA has been shown to find rotational dynamics even if they do not exist (14). Contrastive learning objectives are also known to be affected by the choice of positive and negative views (15). These examples show that, while more expressive models can sometimes improve interpretation, if left unbounded, these models can also lead to results that are difficult to interpret because they start to reproduce too much. When data violates our assumptions of simplicity, we may be susceptible to hallucinations through the kinds of mechanisms illustrated by Shinn (2).

Regardless of how we do dimensionality reduction, if the assumptions and biases underlying a method are not understood then it can be possible to see things in the data that aren’t there. We have already seen how even simple properties of data, like that it changes slowly over time and space, a near universal aspect of the world, means that an algorithm maximizing variance will extract fourier-like oscillatory components. Plotting high-variance components against one another will often have apparently interpretable shapes. But the world is full of such universal and, arguably, weird data structures: local outliers, spatiotemporal smoothness, shifts in time, compositionality, and even the presence of agents who optimize for their own goals. Interpreting the results of dimensionality reduction methods is a truly daunting task. Any result found with dimensionality reduction is compatible with a large family of potential realities.

## Towards Acknowledging Complexity and Ways of Handling it

Neuroscientists often aim to artificially produce simplicity in neural activity by utilizing simple tasks. However, even the simplest of tasks is embedded within a much broader behavioral context, and in reality, animals dynamically switch between the many tasks needed to survive in a complex world, using a wide variety of cues in order to succeed in their environment. Consequently, there is an emerging approach to neuroscience that actively looks for neural representations of unconstrained real-world behavior, a trend in parts enabled by modern computer-vision-based tracking approaches (16, 17) and automated tools for behavior analysis (18, 19). In complex real-world scenarios, data are not simple, and we should not expect any dimensionality reduction algorithm to reveal a single set of factors that are easy to interpret.

So what should we do when faced with complex and real-world behavior? Do we try to find a model with just the right amount of complexity? Look for simpler interpretations within complex models. One radical alternative is to give up on the notion of finding a simple explanation for complex data (20). If the data are complicated, we may still be able to build a model that does justice to it, for example, by building foundation models that are trained on many diverse tasks, across many animals and individuals, and across different sources of data (21–23). Such models can then readily model data complexities because they have a large number of parameters that are tuned on very large datasets. This approach promises to offer many of the benefits of dimensionality reduction—for instance, enabling better decoding—while allowing dealing with data that is not simple or easy to explain. However, there is nothing simple nor interpretable in these models—prediction is much easier than interpretation.

That being said, the kind of large models that can describe real-world behavior may not ultimately be interpretable in the sense that neuroscientists are looking for. The Shinn paper (2) already shows that even with smaller and simpler models, we may see things in the data that aren’t there. In most cases, there may not be a simple way of describing the complexity of the data and models in ways that humans can readily interpret. This leads us to, arguably, the most challenging topic in neuroscience. If brain function doesn’t obey a simple set of principles, what can we understand about it, and how can we navigate the balance between faithfulness to the data and the desire to have simple explanations?
